# Antibiotics alone versus appendectomy to treat uncomplicated acute appendicitis in adults: what do meta-analyses say?

**DOI:** 10.1186/s13017-015-0046-1

**Published:** 2015-10-31

**Authors:** Leonardo Lima Rocha, Felipe Martin Bianco Rossi, Camila Menezes Souza Pessoa, Flavia Nunes Dias Campos, Carlos Eduardo Fonseca Pires, Milton Steinman

**Affiliations:** Intensive Care Unit, Hospital Israelita Albert Einstein, São Paulo, Brazil; Telemedicine Service, Hospital Israelita Albert Einstein, Av. Albert Einstein, 2 andar, bloco D, São Paulo, CEP: 05651-901 Brazil; Surgery Department, Hospital Israelita Albert Einstein, São Paulo, Brazil

**Keywords:** Appendicitis, Appendectomy, Antibiotics, Conservative, Surgery, Meta-analysis

## Abstract

**Background:**

Primary appendectomy is the current standard of care for treating uncomplicated acute appendicitis, but interest in conservative treatment with antibiotics alone has been increasing in recent years. Clinical trials so far have shown controversial results.

**Methods:**

A series of meta-analyses were reviewed. Studies comparing surgery versus antibiotics alone for treating uncomplicated acute appendicitis in adults were included. Descriptive statistics and data on treatment effects were retrieved and summarized.

**Results:**

The conservative approach has a success rate of around 60 % and is associated with shorter pain duration, reduced analgesic medication, faster resolution of the inflammation process, lower expenses and quicker return to work. On the other hand, medical treatment leads to high (up to 20 %) readmission rates and more often requires surgery. An operative approach is associated with higher treatment success rates (>90 %) and very a low mortality rate.

**Conclusion:**

Based on the current body of evidence, the use of antibiotics for primary treatment of uncomplicated acute appendicitis cannot be routinely recommended. Appendectomy remains the gold-standard treatment.

## Introduction

Acute appendicitis is the most common cause of inflammatory acute abdomen [1]. The incidence of acute appendicitis varies from 250,000 to 280,000 cases per year in the United States, which accounts for more than 1 million hospital days per year [2] and a cost of more than 3 billion dollars [3]. For more than a century, acute appendicitis has been treated by surgery, i.e., appendectomy [4, 5], with mortality rates as low as 0.07 to 0.7 % [6, 7]. Nevertheless, surgical intervention is associated with greater pain, adherence and hernia development, ileus, venous thromboembolic events, cardiopulmonary complications and increased costs.

During the 1950s, an initial non-operative approach for acute appendicitis was attempted, but it was not generally accepted at the time [8]. Appendicitis complicated by appendicular abscess/phlegmon may be managed with antibiotics and non-operative strategies with reduced complication rates compared to surgery [9], like other acute inflammatory intestinal conditions – i.e. diverticulitis and enterocolitis [10]. In this sense, antibiotic therapy may be associated with reduced costs of treatment, avoiding operation and its consequent complications. However, the use of antibiotics alone as primary therapy for uncomplicated acute appendicitis is still being assessed, and studies have shown conflicting results so far [11, 12].

The aim of this study is to review available meta-analyses comparing antibiotics alone versus appendectomy for uncomplicated acute appendicitis in adults.

## Materials and methods

### Study selection

We included all meta-analyses with retrospective or prospective observational and/or experimental studies and compared clinical (antibiotics alone) versus surgical (open or laparoscopic appendectomy) treatment for suspected uncomplicated acute appendicitis in adults (>18 years-old). Uncomplicated acute appendicitis was defined as acute inflammation of the appendix in the absence of an abscess, phlegmon, free perforation or peritonitis. We excluded meta-analyses enrolling patients with complicated appendicitis, without quality evaluation of individual studies, and children.

### Search strategy

We searched MEDLINE, Embase, and The Cochrane Library databases up to June 2015 for meta-analysis in adults without language restrictions. The following MeSH (Medical Subject Headings) terms were used: “appendicitis”, “meta-analysis”, “appendectomy”, and “anti-bacterial agents” with Boolean terms. The “related article” function and article references were searched to add other eligible meta-analyses. Experts in the field were also consulted for suggestions on further studies.

### Data extraction

Two different authors (L.L.R. and F.M.B.R.) independently performed the search and retrieved eligible meta-analyses based on previously set inclusion and exclusion criteria. In case of no two authors consensus, a third one (M.S.) was contacted. The data extracted from each study included: first author, year of publication, geographic region, number and design of studies included, inclusion and exclusion criteria, studied population characteristics, statistical analysis (e.g. data on treatment effect, random and/or fixed models; heterogeneity), primary and secondary outcomes and study limitations.

### Statistical analysis

Descriptive statistics summarizing the included studies were calculated. The mean ± standard deviation was used for normally distributed continuous variables. The median and interquartile range was used for non-normally distributed continuous variables. Odds ratio and 95 % confidence interval were calculated for specific outcomes from data available in the individual studies whenever not directly calculated by the meta-analysis authors. The meta-analysis pooled results, including the measures of central tendency and measures of treatment effect plus their associated 95 % confidence interval (CI) and *p*-value when available, were extracted and summarized. Ratios were calculated between appendectomy and antibiotics alone groups whenever applicable.

## Results

A total of eight meta-analyses were retrieved [11, 13–19] and their general characteristics are shown in Table 1. The mean number of pooled patients included in each meta-analysis was 862 ± 211, which accounts for a mean of 403 ± 74 patients in the primary antibiotic therapy group and a mean of 458 ± 163 patients in the primary appendectomy group. The mean number of studies included was 4.38 ± 1.07. Generally, the quality of the individual studies was deemed low to moderate by different scales. From all the meta-analyses included, there were eight different individual studies, in different combinations [20–27], with the majority being prospective studies (87.5 %). Only one meta-analysis included a retrospective study [23].

**Table 1:** Characteristics of included meta-analyses comparing antibiotics alone versus appendectomy in patients with acute uncomplicated appendicitis

Author (year)	Patients included			Study included	Study design	Quality evaluation
	Total	A	S			
Varadhan et al. (2010) [13]	661	350	311	3 [24–26]	RCT	2.7^a^
Liu et al. (2011) [14]	1201	433	768	6 [24–29]	RCT	≥5^b^
					Observational	
Ansaloni et al. (2011) [15]	741	390	351	4 [24–26, 28]	RCT	Poor
Wilms et al. (2011) [16]	901	415	486	5 [25, 26, 28–30]	RCT	Low-moderate
Mason et al. (2012) [17]	980	510	470	5 [24–26, 28, 30]	RCT	1.8^a^
Varadhan et al. (2012) [18]	900	470	430	4 [24–26, 30]	RCT	Moderate^c^
Liu et al. (2014) [19]	983	391	592	5 [24–26, 29, 31]	RCT	3.2^a^
Kirby et al. (2015) [11]	531	268	263	3 [25, 26, 30]	RCT	N/A

^a^Mean Jadad score

^b^NOQAS

^c^GRADE system

*Abbreviations*: *A* antibiotics therapy group, *S* surgery group, *RCT* randomized controlled trial, *NOQAS* Newcastle-Ottawa Quality Assessment Scale, *GRADE* Grading of Recommendations Assessment, Development and Evaluation, *N/A* data not available

The main outcome metrics of each meta-analysis are illustrated in Table 2. Conservative therapy with antibiotic therapy was associated with significantly reduced minor (i.e. superficial wound infection, prolonged postoperative course, diarrhea, *Clostridium difficile* infection, fungal infection, etc.) and major (i.e. abscess formation, peritonitis, deep wound infection, reoperation, small bowel obstruction, postoperative cardiac events and venous thromboembolism) complication rates in most meta-analyses. The exception was the study by Kirby et al. [11], which reported a risk ratio of 7.71 (95%CI 2.33, 25.53) for major complications (i.e. peritonitis or abscess after the primary intervention) in patients assigned to antibiotic groups.

**Table 2:** Measured outcomes from different meta-analyses comparing appendectomy versus antibiotics alone for acute uncomplicated appendicitis

Outcome	Measure of Effect (95 % CI)	*p*-value	Heterogeneity	Authors’ conclusion
Complications	0.43 (0.15, 1.21) [13]	0.11	Moderate	
	0.31 (0.19, 0.49) [14]	<0.05	Not present	
	1.92 (1.30, 2.85) [15]	N/A	Not present	
	0.83 (0.72, 0.91) [16]	N/A	Low	Favors antibiotics
	0.54 (0.37, 0.78) [17]	0.001	Moderate	
	0.69 (0.54, 0.89) [18]	0.004	Not present	
	0.86 (0.59, 1.26) [19]	0.44	Not present	
Treatment efficacy	4.54 (3.02, 6.82) [13]	<0.001	N/A	
	6.01 (4.27, 8.47) [15]	N/A	Not present	Favors surgery
	Crosses NI margin [16]	N/A	N/A	
	8.89 (5.94, 13.32) [18]	<0.001	N/A	
Treatment failure	6.9 % ± 4.4 % [14]	N/A	N/A	Favors surgery
	6.72 (3.48, 12.99) [17]	<0.001	Moderate	
Readmissions	15 % [13]			
	14.2 ± 10.6 % [14]			Favors surgery
	20 % [18]			
Complicated appendicitis	0.46 (0.19, 1.12) [18]	0.09	High	
	0.58 (0.18, 1.90) [18]	0.37	Moderate	Inconclusive
	0.73 (0.29, 1.84) [15]	N/A	Not present	
Pain/Analgesia	ATB less pain [15]	<0.001	N/A	
	−1.55 (−1.96, −1.14) [17]	<0.001	Not present	Favors antibiotics
	−0.13 (−0.28, 0.03) [17]	0.11	Low	
Length of hospital stay	0.11 (−0.22, 0.43) [13]	0.53	Moderate	
	0.66 (0.44, 0.87) [15]	<0.001	Low	
	0.34 (−0.06, 0.73) [17]	0.09	Low	Inconclusive
	0.34 (−0.19, 0.87) [18]	0.20	Low	
	0.01 (−0.01, 0.03) [19]	0.26	High	
Time to return to work	−0.19 (−0.33, −0.06) [17]	0.005	Not present	Favor antibiotics
	−5.20 (−6.99, −3.40) [19]	<0.001	High	

*Abbreviations*: *ACI* confidence interval, *ATB* antibiotics, *NI* non-inferiority, *N/A*, data not available

The conservative treatment was associated with faster recovery from inflammatory response, as evaluated by a better temperature curve, neutrophils count and C-reactive protein levels. Also, patients treated with antibiotics alone experienced significantly less pain duration and analgesic medication consumption. Regarding the return to daily activities, patients treated with antibiotics alone had a faster return to work (WMD, −5.20 95%CI −6.99, −3.40 days; *p* < 0.001) and less sick leave duration (MD, −0.19 95%CI −0.33, −0.06; *p* = 0.005). At the 1-year follow-up, there was no significant difference between groups in terms of perforation rates (10.6 % versus 9.3 %; *p* = NS) [11].

Comparing treatment efficacy, appendectomy was significantly more efficient than antibiotics alone (OR, 6.01 95%CI 4.37, 8.46) when overall treatment was analyzed. Comparing cure up to 1 year, the comparison was inconclusive according to a 20 % non-inferiority margin set by Wilms et al. [16] For the first 24 to 48 h (6.9 % versus 7.3 %) and initial hospitalization (OR, 2.43 95%CI 0.94, 6.33; *p* = 0.07), both treatments were equivalent in terms of treatment failures. On the other hand, exclusive antibiotic therapy was associated with a higher rate of readmission, which varied between 14.2 and 20 %. And from these readmissions, the absolute majority of patients were treated by the surgical approach, with a second course of antibiotics used in only a few cases (data not shown).

In the majority of meta-analyses, the length of hospital stay was not significantly different between the two groups (Table 2). The exception was the meta-analysis by Wilms et al. [16], which concluded that patients submitted to appendectomy had a reduction of 34 % in their length of hospital stay compared to conservative therapy (OR, 0.66 95%CI 0.44, 0.87).

The meta-analysis by Ansaloni et al. [15] was the only one that addressed costs. In this study, conservative therapy was associated with a mean cost reduction of USD 1257 per patient treated (USD 2893 versus USD 4150).

## Discussion

To the best of our knowledge, this is the first study to assess a series of meta-analyses comparing appendectomy to antibiotics for uncomplicated acute appendicitis. The results of our review of this series of meta-analyses showed that conservative treatment with antibiotics for uncomplicated acute appendicitis is associated with less complications, faster recovery from an inflammatory state, shorter pain duration and reduced consumption of analgesic medication, faster return to work and reduced costs. Conversely, conservative treatment is associated with significantly higher treatment failure and readmission rates compared to primary appendectomy.

Treating appendicitis implies understanding acute appendicitis as a spectrum of disease, ranging from mild spontaneous resolution cases (up to 20 %) [28] through perforation and generalized peritonitis. In this sense, the actual challenge is to distinguish those patients who will spontaneously resolve the inflammatory state from those who will develop complications (gangrene, abscess, perforation and peritonitis). Some studies attempted to determine the risk factors for complicated appendicitis [29–31]. The main risk factors associated with complicated appendicitis are clinical (i.e. male sex, age ≥60 years of age and onset of symptoms) and laboratorial (i.e. leukocytosis, elevated C-reactive protein and bilirubin). Imaging investigations may improve diagnosis, avoiding conservative treatment of patients with complicated appendicitis [32].

CT scan, US and clinical exam, or a combination of these were used to diagnosis uncomplicated acute appendicitis. The CT scan in acute appendicitis has the highest diagnostic sensitivity and specificity, [28] increasing the appendectomy rate, especially in patients with lower Alvarado score [33, 34]. On the other hand, it is related to radiation exposure. The use of combined clinical scores (i.e. Alvarado score and Appendicitis Inflammatory Response score) allied with rational use of imaging methods (i.e. no routine use of CT scan, US in most cases) showed to be nearly as reliable as CT scan for diagnosis of acute appendicitis [35].

Antibiotics success rates as primary therapy for uncomplicated acute appendicitis varied from 58.3 to 73.4 %. As much as 42 % of patients primarily treated with antibiotics will require appendectomy later on in the disease’s course [13]. Treating patients with uncomplicated appendicitis initially with antibiotics is safe even if an appendectomy is required later [36]. The use of prophylactic antibiotics during appendectomy is associated with a 3-fold reduction in the incidence of wound infection after appendectomy [37]. In the included studies, the surgical wound infection rate in patients undergoing primary appendectomy was 2.8 % when antibiotic prophylaxis was employed and 11.8 % when it was not [20, 26]. This may have introduced a bias in the meta-analyses that used wound infection as a primary outcome [18], since studies that did not use prophylactic antibiotics may be prone to favor the conservative treatment group [21]. Another issue is the use of heterogeneous antibiotic schemes, which includes oral, intravenous or mixed courses and mono/multi drug therapy. Some antibiotic schemes, such as amoxicillin plus clavulanic acid for Escherichia coli, may be ineffective for treating common gastrointestinal tract bacteria.

Open appendectomy, albeit largely performed worldwide, is associated with longer pain duration and analgesic consumption, longer time to return to work and sick leave, and higher rates of wound infection compared to laparoscopic appendectomy [38]. Patients were submitted to open appendectomy in the majority of studies included. This may have contributed to the significant differences in pain and analgesic consumption, time to work and wound infection rates observed between antibiotics and surgery groups.

All the studies suffered from several methodological limitations and the majority of the outcomes studied in individual meta-analyses presented some degree of heterogeneity, making it challenging to draw definitive conclusions. The study by Hansson et al. [20] showed a very high crossover rate (47.5 %) mainly from antibiotics to the appendectomy group, this might have introduced bias to meta-analyses, which includes this study. Due to the reduced number of enrolled patients, no subgroup analyses were performed to account for possible confounders such as sex, age, diagnostic assessment, surgical procedure (open versus laparoscopic), and use of prophylactic antibiotics to cite a few. Additionally, unblinded outcome assessment may overestimate the effects of treatment and lack of allocation concealment may have introduced selection bias to the analysis.

The recently published APPAC randomized clinical trial [36] was a well-designed adequately powered non-inferiority study comparing antibiotics (ertapenem for 3 days plus levofloxacin and metronidazole for 7 days) to appendectomy. It included in the primary analysis 272 patients in the appendectomy group and 256 in the antibiotic therapy group. The primary endpoint for surgery (successful completion of appendectomy) occurred in 99.6 % (95 %CI 98 %, 100 %) and the primary endpoint for antibiotic therapy group (discharge from hospital without need for appendectomy and no recurrent appendicitis within 1-year follow-up) occurred in 72.7 % (95 %CI 66.8 %, 78 %). The difference between treatments was −27 % (95%CI −31.6 %, ∞; *p* = 0.89), which crossed the non-inferiority margin of 24 %. The rate of complicated appendicitis did not differ between groups, and the antibiotics group presented lower complication rates. The length of stay was significantly higher in the antibiotics group, though not clinically significant. The ASAA (www.clinicaltrials.gov, NCT01421901) is another well-designed study whose findings will be published soon. The inclusion of these studies in future meta-analyses will help improve the methodological quality and robustness of the results.

## Conclusion

Appendectomy is considered the gold standard for treating uncomplicated acute appendicitis. Nevertheless, for a selected subgroup of patients with no risk factors for complicated appendicitis and/or high surgical risk, conservative therapy with antimicrobials may be safe and effective. The decision to treat patients with acute uncomplicated appendicitis must be made on an individual basis and these patients may be followed closely.

## Abbreviations

CI: Confidence interval
CT: Computed tomography
GRADE: Grading of Recommendations Assessment, Development and Evaluation
LOS: Length of stay
MD: Mean difference
N/A: Data not available
NI: Non-inferiority
NS: Not statistically significant
NOQAS: Newcastle-Ottawa Quality Assessment Scale
OR: Odds ratio
RCT: Randomized controlled trial
RR: Risk ratio
US: Ultrasonography
WMD: Weighted mean difference
